# Fine-tuning *ab initio* XANES spectra calculations using the Bayesian optimization algorithm

**DOI:** 10.1107/S1600577526006430

**Published:** 2026-07-28

**Authors:** Andrey A. Sapronov, Dmitry E. Doronkin, Jan-Dierk Grunwaldt

**Affiliations:** a Institute of Catalysis Research and Technology Karlsruhe Institute of Technology Hermann-von-Helmholtz-Platz1 D-76344Eggenstein-Leopoldshafen Germany; b Institute for Chemical Technology and Polymer Chemistry Karlsruhe Institute of Technology Engesserstraße 20 D-76131Karlsruhe Germany; ESRF – The European Synchrotron, France

**Keywords:** XANES, Bayesian optimization, XANES simulation, *ab initio* calculations, spectra simulations

## Abstract

Theoretical modeling of X-ray absorption near-edge structure (XANES) spectra is often performed to interpret experimental spectra and to relate spectral features to the atomic and electronic structure of materials. The sensitivity of the XANES spectra to coordination environment, oxidation state and structural disorder is explored using *FEFF* and *FDMNES* simulation codes. With a suitable configuration of these instruments, one can also explore the quantitative characteristics of the studied materials, such as lattice constant, nanoparticle geometry, coordination numbers and longer-range ordering. However, fine-tuning the input parameters is often resource-consuming and non-intuitive. We test the application of the Bayesian optimization (BO) algorithm in finding the most optimal simulation parameters for the theoretical XANES spectra and compare the results using different spectrum similarity metrics. The BO method outperforms the random search technique by a factor of three in speed and shows that the correlation-based metrics provide better shape-level agreement than those defined as normalized distance.

## Abstract 2

A Bayesian optimization technique is used to tune the *FEFF* and *FDMNES* packages and to improve matching between theoretical and experimental spectra. The tests were performed on monometallic Ni, Fe and Pd *K*-edge XANES spectra using several different spectrum similarity metrics.

## 1. Introduction

Due to high structural sensitivity at the atomic scale, X-ray absorption spectroscopy (XAS) is widely used for probing local coordination environments, oxidation states and electronic structure in materials ranging from catalysts and battery materials to complex biological systems. X-ray absorption near-edge structure (XANES) is mainly sensitive to the oxidation state and coordination environment, and extended X-ray fine structure (EXAFS) spectroscopy is used primarily to determine coordination numbers, distances and types of the nearest neighbors to the absorbing atom. This is one of the characterization techniques that does not require any local or long-range ordering and can be used on a wide range of substances. Hence, it has become the characterization tool of choice in not only materials research and catalysis but also for molecular species and amorphous materials.

Identification of the species in question mostly relies on analyzing the XANES part of the spectrum. An extensive overview of XAFS analysis tools is given by Bordiga *et al.* (2013 ▸). In the context of analyzing experimental data, one can name four basic methods (Martini *et al.*, 2020 ▸):

(i) Fingerprinting approach, where the features of the experimental spectrum are compared with features in the reference spectra (*e.g.* obtained from spectral libraries or theoretically calculated).

(ii) Gradient descent approach, which uses a series of steps to minimize the difference between experimental and theoretical spectra quantified by some objective function. This method is used in such codes as *GNXAS* (Di Cicco & Filipponi, 2009 ▸; Filipponi *et al.*, 1995 ▸; Filipponi & Di Cicco 1995 ▸), *Viper* (Klementev, 2001 ▸), *MXAN* (Benfatto *et al.*, 2021 ▸; Benfatto *et al.*, 2001 ▸) and *IFEFFIT* (Ravel & Newville, 2005 ▸).

(iii) Indirect approach for modeling the difference between experimental and theoretical spectra with a multidimensional polynomial function, with the minimal discrepancy being found using interpolation techniques. Such a method was applied in the program *FitIt* (Smolentsev & Soldatov, 2007 ▸) and its successor *pyFitIt* (Martini *et al.*, 2020 ▸).

(iv) Direct approach, which solves the inverse problem of parameterizing structural parameters to fit the XANES spectrum descriptors. This method embraces a variety of machine-learning-based methods, largely neural networks, which are used to build a black-box correspondence between the XAS spectrum and the system structure (Timoshenko & Frenkel, 2019 ▸; Torrisi *et al.*, 2020 ▸).

The X-ray absorption spectrum simulation plays an important role in the XAS data analysis, helping in qualitative and quantitative interpretation of the measurements. In the context of characterization of heterogeneous (photo-/electro-)catalysts, we are often interested in analyzing solid transition metal nanoparticles (NPs). While spectra of monometallic NPs can often be fitted using library reference spectra of corresponding metal foils or model well defined samples, the situation becomes more complicated for samples containing several metals capable of forming alloys, small particles and/or intermetallic compounds, for which experimentally obtained reference spectra often do not exist and need to be calculated. While the phenomena and the related processes are generally understood, the efforts to provide a precise simulation of EXAFS and especially XANES spectra are still ongoing. There are several simulation codes aimed at the calculation of XANES spectra and their parameters. The *FEFF* package (Rehr *et al.*, 2010 ▸; Kas *et al.*, 2021 ▸) uses real-space Green’s function and multiple scattering for the calculation of excitation spectra and electronic structure. Another tool, *FDMNES*, can solve the Schrödinger equation using full-potential finite-difference to evaluate near-edge structure (Bunău & Joly, 2009 ▸). The codes *OCEAN* (Vinson *et al.*, 2011 ▸) and *exciting* (Gulans *et al.*, 2014 ▸) implement first-principles calculations based on both ground-state density functional theory (DFT) and the Bethe–Salpeter equation. In addition to this, *ORCA* (Neese *et al.*, 2020 ▸) has attracted a lot of attention as a general-purpose package with a special focus on transition metals and spectroscopy, featuring DFT, wavefunction-based correlation, semi-empirical and force-field methods.

*FEFF* and *FDMNES* are the tools that are commonly used for XAS spectrum simulation due to their ease of use, computational speed and out-of-the-box results quality. Our work is therefore focused on these two codes.

*FEFF* is a widely used *ab initio* simulation package for XAS, capable of calculating both XANES and EXAFS spectra based on real-space multiple scattering theory. It models the propagation of the photoelectron excited from a core level in the potential created by the surrounding atomic environment, using Green’s function method and self-consistent potentials. *FEFF* supports a wide range of features, including polarization effects, Debye–Waller factors for thermal disorder and many-body corrections for inelastic losses and core-hole effects. Its cluster-based approach makes it particularly well suited for systems with local disorder, surfaces and finite-size effects.

*FDMNES* is another *ab initio* simulation package designed to calculate XANES and EXAFS (with the help of the *FDMX* extension). It solves the Schrödinger or Dirac equation for a photoelectron in a full-potential framework using either a finite difference method or multiple scattering formalism. *FDMNES* supports both spherical and non-spherical potentials, allowing accurate treatment of low-symmetry environments, anisotropic systems and complex electronic structures. The code accounts for core-hole effects, spin–orbit coupling and electric quadrupole transitions, making it particularly powerful for simulating detailed near-edge features and dichroism.

The XAS simulation codes employ a range of physical models to represent how the X-rays interact with the matter and how this interaction is influenced by the electronic and atomic structure around the absorbing atom. The main physical phenomena described by these models include the photoelectric effect, multiple scattering of the photoelectron, unoccupied density of states and core-hole effects. Not all of these models are first-principle ones and many require being configured with tunable, often empirically found, parameters. Being used by non-experts in the underlying implementation, the XAS simulation codes are often considered as black-box software with configurations made by intuition or at random. On the other hand, the definitions of the best fit to the experimental measurements are often vague and ambiguous, which makes it hard to identify which parameter set brings a better prediction of the experimental spectrum of a given species.

In the case of complex system optimizations that include resource-intensive calculations and where the explicit dependence on the optimized parameters is not known, there are a handful of methods to determine the best modeling parameters. The most often used are the grid search, random search and Bayesian optimization (BO). The grid search is the most ineffective one, since all possible parameter combinations are trialed to cover the whole domain of possible values, and then the best combination is selected. Random search consists of randomly selecting parameter combinations until there is no improvement for a predefined number of trials. This method is known to outperform grid search in the case of large dimensionality of parameter space (Bergstra & Bengio, 2012 ▸). The BO algorithm (Brochu *et al.*, 2010 ▸) is known to beat these two in the majority of cases by utilizing knowledge about previous trials in order to select the parameter set for the next ones. This method is currently a standard go-to in the contemporary hyperparameter tuning tasks, whether machine learning modeling, physical system optimizations or computer simulations are considered.

The Bayesian techniques were previously used for XAS analysis in different directions. Iwamitsu *et al.* (2020 ▸) used a Bayesian spectroscopy technique to decompose pre-edge peaks from the XANES region of the α-Fe_2_O_3_ Fe *K* spectrum and a similar technique was used (Kashiwamura *et al.*, 2022 ▸) for XANES spectrum deconvolution into the sum of multiple basis functions. The method of two coupled BOs, named adversarial Bayesian optimization (ABO), which comprises an automated parameter fitting to obtain the atomic Hamiltonian and at the same time perform efficient sampling-based active learning, was tested for NiO by Zhang *et al.* (2023 ▸). Although not directly related to optimization, Haddad *et al.* (2024 ▸) used Bayes’ theorem to estimate the structural parameter correlations in the EXAFS analysis and demonstrate its application by building structural models of CdS magic-size clusters using EXAFS data. Finally, the BO method was applied in the grazing exit X-ray absorption near-edge structure (GE-XANES) experiments to maximize the efficiency of the energy scanning and significantly reduce the measurement time (Cakir *et al.*, 2024 ▸).

In this work, we apply the BO algorithm to fine tune the XAS simulation data from *FEFF* and *FDMNES*. We test an approach similar to the gradient descent, but without relying on differentiability. We consider the deviation of the modeled spectra from the measured ones as a black-box objective function, with hyperparameters defined by the simulation package control cards. Crucially, this study includes an evaluation of several spectrum similarity metrics when applied to matching the experimental spectra of several transition metals commonly explored in relation to catalysis and energy storage applications.

## 2. Methods

### 2.1. Optimized parameters

Table 1 ▸ provides a list of *FEFF* (v10.0.0) package input parameters that influence the XANES/EXAFS spectrum simulation. For our study, we chose to optimize the prediction of the XANES spectrum. The same method in principle can be applied to the EXAFS spectra too, given that the corresponding parameters are included and the control cards are adjusted (Rehr *et al.*, 2020 ▸).

A similar list of configuration parameters for the *FDMNES/FDM* (v2025_07_11) package is provided in Table 2 ▸.

### 2.2. Objective functions for BO

The method of BO relies on prior knowledge about the objective function and its properties, such as smoothness. It therefore operates in the functional space by employing the Bayes’ theorem, which helps to direct the sampling, and to manage the trade-off between exploration and exploitation of the search space. If **x**_*i*_ is the *i*th sample and *f*(**x**_*i*_) is the value of the loss function at **x**_*i*_, then the accumulated observations are 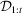 = 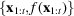. As we accumulate the observations, the prior distribution is combined with the likelihood function 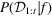. Under the assumption that the objective function is smooth and noise-free, the components are combined to obtain the posterior distribution 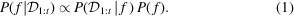
The objective function is thus interpreted as a surrogate function formally described as a posterior mean function of a Gaussian process. The next sampling location **x**_*t*+1_ is determined using the acquisition function which provides a ‘hint’ where the resulting observation will have either the highest probability of improvement, or Bayesian expected losses, or satisfy another criterion built into the acquisition function definition.

When performing parameter optimization, the definition of the spectrum similarity metric in the loss function is important. Since the experimentally measured XAS spectra are unitless, and the theoretical predictions may have arbitrary spectrum normalization, the most relevant metrics are deemed those that are less sensitive to the magnitude and more sensitive to the distribution of shape and peak positions. One can consider the following metrics (with details given in Appendix *A*[App appa]) to quantify how well the simulated spectra fit to the experimental data: L2 normalized distance, *R* factor, cosine similarity, Pearson correlation coefficient, Spearman rank correlation score. These metrics display different sensitivities to shape and absolute discrepancy. The L2 normalization distance is constructed as the quadratic norm of the difference between two unit vectors along the direction of intensity vectors (*i.e.* vectors with coordinates being the intensity values of the spectra, assuming the energy values are the same). The *R* factor is a ratio of the difference between theoretical and experimental intensity vectors and the experimental one, and cosine similarity is a cosine angle between the theoretical and experimental intensity vectors. Correlation coefficients capture the mutual trends in the spectral shape. To distinguish between normalization of XAS spectra, which involves background subtraction, pre-edge and post-edge fitting, *etc*. and normalization used in metric computation, we will refer to the former as edge-step normalization and to the latter as vector normalization.

### 2.3. Experimental spectra

**Figure 1:**
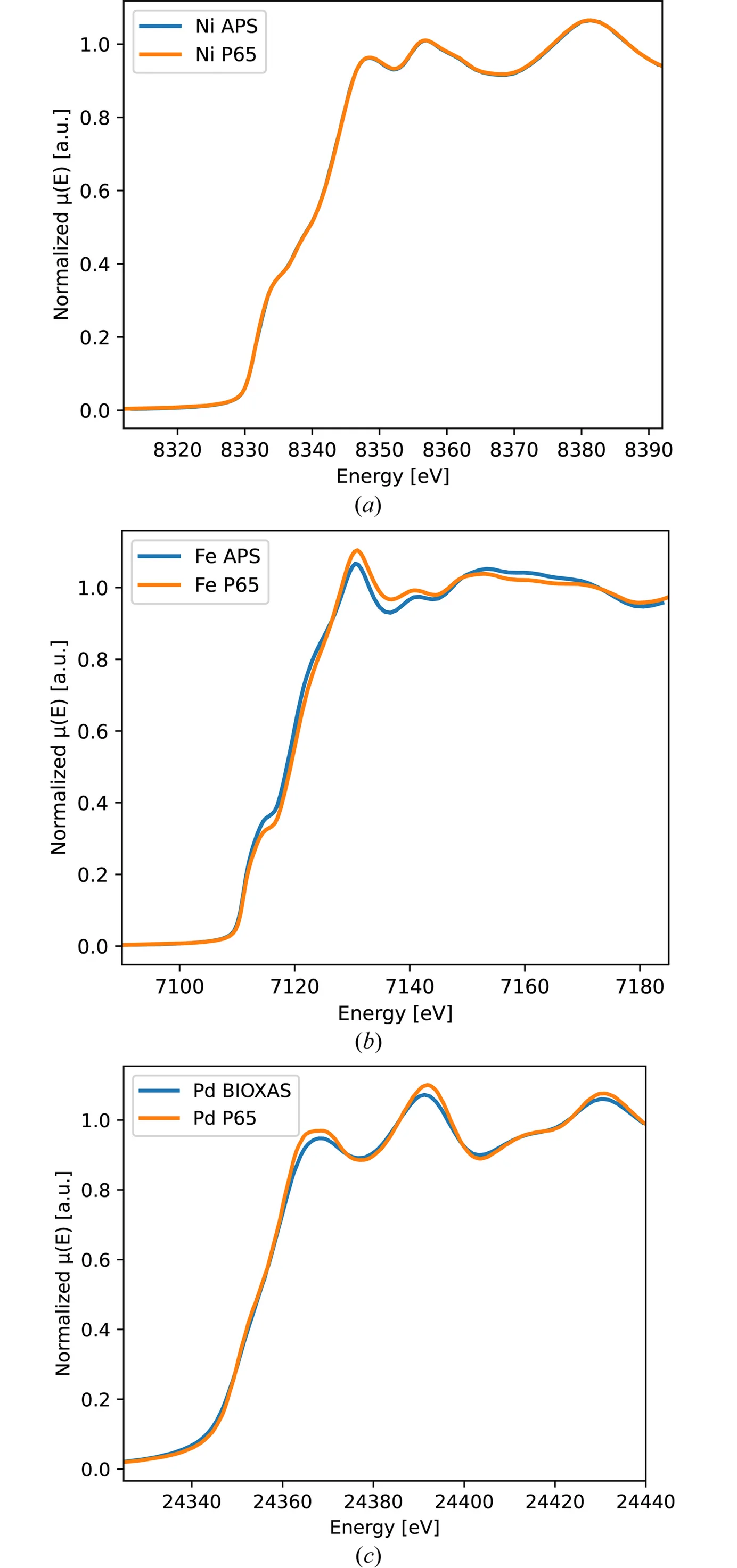
XANES region of the experimentally measured at the (*a*) Ni *K*-, (*b*) Fe *K*- and (*c*) Pd *K*-edge XAS that were used for the analysis

The *K*-edge transmission spectra of reference Ni, Fe and Pd metal foils used in this study are listed in Table 3 ▸ and their XANES regions are presented in Fig. 1 ▸.

These measurements come from different facilities: 13-ID-C,D (APS, 2025*b* ▸) and 13-BM-D (APS, 2025*a* ▸) beamlines of the Advanced Photon Source (APS), BioXAS spectroscopy beamline 07ID-2 (CLS, 2025 ▸) of the Canadian Light Source (CLS) and P65 beamline of the PETRA III synchrotron (Welter *et al.*, 2019 ▸). The datasets in the XDI format (Ravel & Newville, 2016 ▸) are publicly available in the following databases: X-ray Absorption Data Library (XASLIB, 2002 ▸), CLS XAS Database (Spasyuk, 2025 ▸) and RefXAS (Paripsa *et al.*, 2024 ▸) and their corresponding sample ids are shown in Table 3 ▸. P65 samples were recorded by the authors using reference foils available at the beamline. These spectra were preprocessed using standard energy alignment and edge-step normalization procedures in the *Larch* (Newville, 2013 ▸) package and in-house-developed Python scripts (XASProc, 2025 ▸). The preprocessing parameters for each metal are shown in Table 4 ▸.

There are slight differences visible for Fe and Pd spectra, possibly related to the different foil quality (suspected for the case of Fe foils), energy resolution (suspected for the case of Pd foils) and the preprocessing of the respective spectra. Different facilities and beamlines have different beam parameters, such as flux, energy resolution, fraction of higher-order harmonics in the beam and others, as well as different detection modes and systems, which influence the resulting spectral features (Chantler *et al.*, 2018 ▸), and thus may impede the optimization convergence or lead to different optimal parameters of the simulation.

### 2.4. Optimizing the spectra simulation

The optimization flow was implemented in a Python program *BO-XAS* (BO-XAS, 2025 ▸). The code is interfaced with Optuna (Akiba *et al.*, 2019 ▸) and uses the *ASE* (Hjorth Larsen *et al.*, 2017 ▸; Bahn & Jacobsen, 2002 ▸) ecosystem to simulate the crystal structure. The program needs a local *FEFF* or *FDMNES* installation, experimental spectra as XDI files and a configuration file. The optimization steps and visualization were run using *Jupyter notebooks* (Kluyver *et al.*, 2016 ▸). In the optimization process, the headers of the input files for the *FEFF* or *FDMNES* simulation packages were created from predefined templates with parameters substituted for every iteration. With simulation time strongly dependent on the parameter values (essentially for the radii) the maximum values were limited, as shown in Table 5 ▸. The convergence of the self-consistent field (SCF) is a vital prerequisite for adequate XAS spectrum simulation in *FEFF* and *FDMNES*. Therefore, lower limits were also set on the corresponding radii *R*_SCF_ and *R*_self_, justified in Appendix *B*[App appb]. The parameter *V*_*r*0_ in the EXCHANGE card, adjusting the exchange correlation potential which adds a constant shift to the Fermi level, gives a linear shift of the resulting spectrum along the energy axis. This parameter is typically used to account for the energy offset relative to the experimental measurement. In the case of algorithmic optimization, the energy calibration part can be moved into the objective function, casting out one whole dimension. The imaginary part of the potential *V*_*i*0_ influences the broadening and is kept to account for the instrument-related effects in the spectra.

In order to produce the simulated spectra for particular species, both *FEFF* and *FDMNES* also require the atomic structure to be defined in their input files. In this work, we used the *ASE* library (Hjorth Larsen *et al.*, 2017 ▸) to build the monometallic crystal structures of the samples described above. For Ni and Pd samples, we used face-centered structures with lattice constants of 3.524 Å and 3.890 Å, respectively, and, for Fe samples, we used a body-centered structure with a lattice constant of 2.866 Å (CRC Handbook, 2004 ▸). The cubic bulk cell was generated for every element and then either converted to a crystal description for *FDMNES* or used to produce explicit cluster geometry for *FEFF*.

One of the popular open-source libraries that implements the BO algorithm is the Optuna library (Akiba *et al.*, 2019 ▸), which was used in the current study. Optuna is an open-source Python framework that provides a flexible interface to define arbitrary objective functions, supports efficient sampling strategies such as tree-structured Parzen estimator (Bergstra *et al.*, 2011 ▸) and Covariance Matrix Adaptation Evolution Strategy (CMA-ES) (Hansen & Ostermeier, 2001 ▸), and includes pruning mechanisms for early stopping of unpromising trials. The algorithm was configured to perform a series of objective function evaluations varying the input parameters and to stop after reaching a limit of non-improving trials. The stability tests show that ten trials for this limit are enough to reach the objective minimum when fitting the experimental XANES spectrum with *FEFF* or *FDMNES*.

## 3. Results

**Figure 2:**
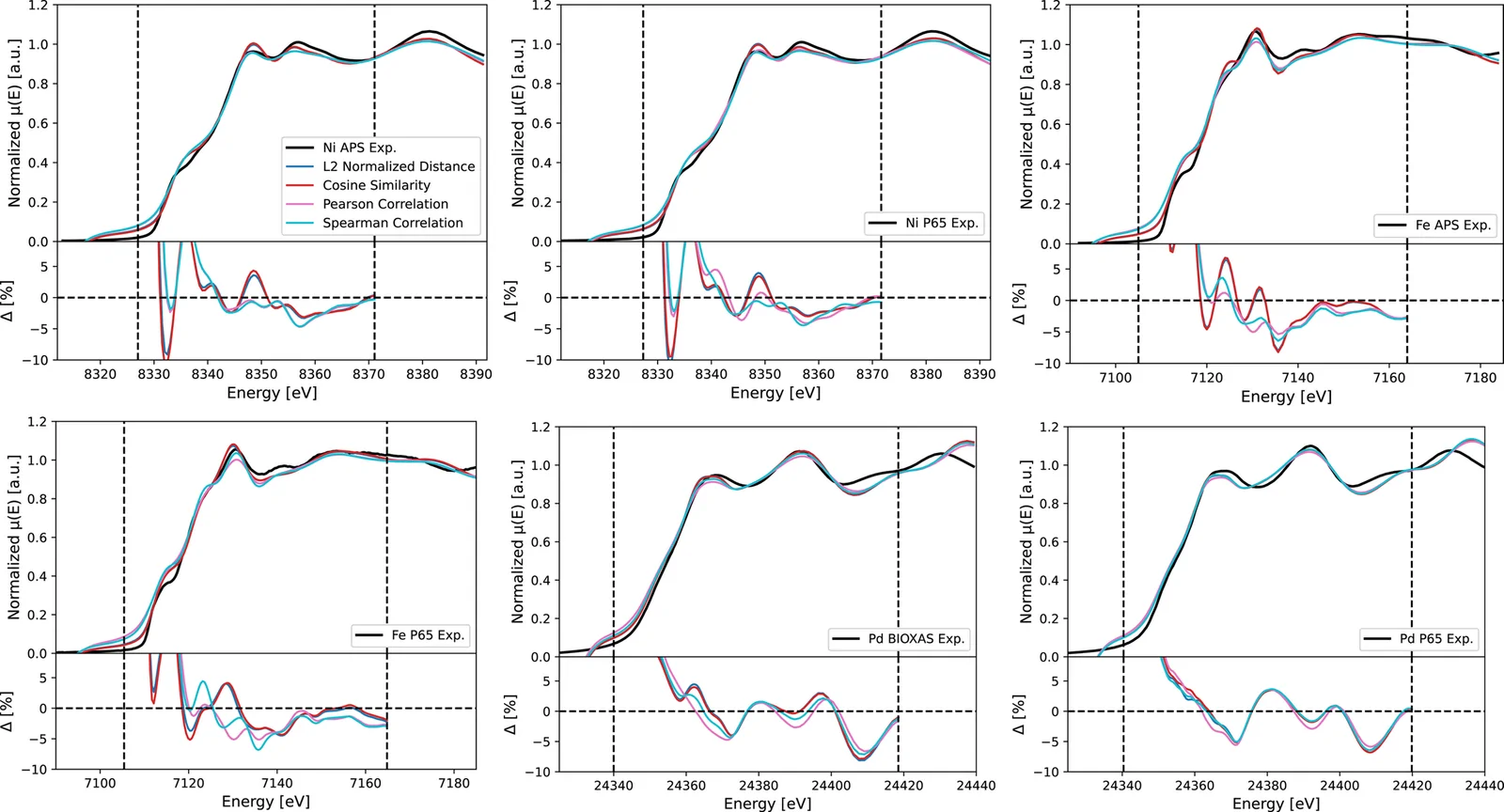
Results of approximating experimental spectra of different metals with their respective simulations using the *FEFF* package. The upper section shows the edge-normalized experimental μ_exp_ and simulated μ_th_ XANES spectra, the lower section shows the relative difference between the simulated spectrum and experimental one Δ = (μ_th_ − μ_exp_)/μ_exp_ × 100%. Vertical dashed lines denote the fitted energy range. The color legend for simulated spectra in the first plot applies to all other plots.

**Figure 3:**
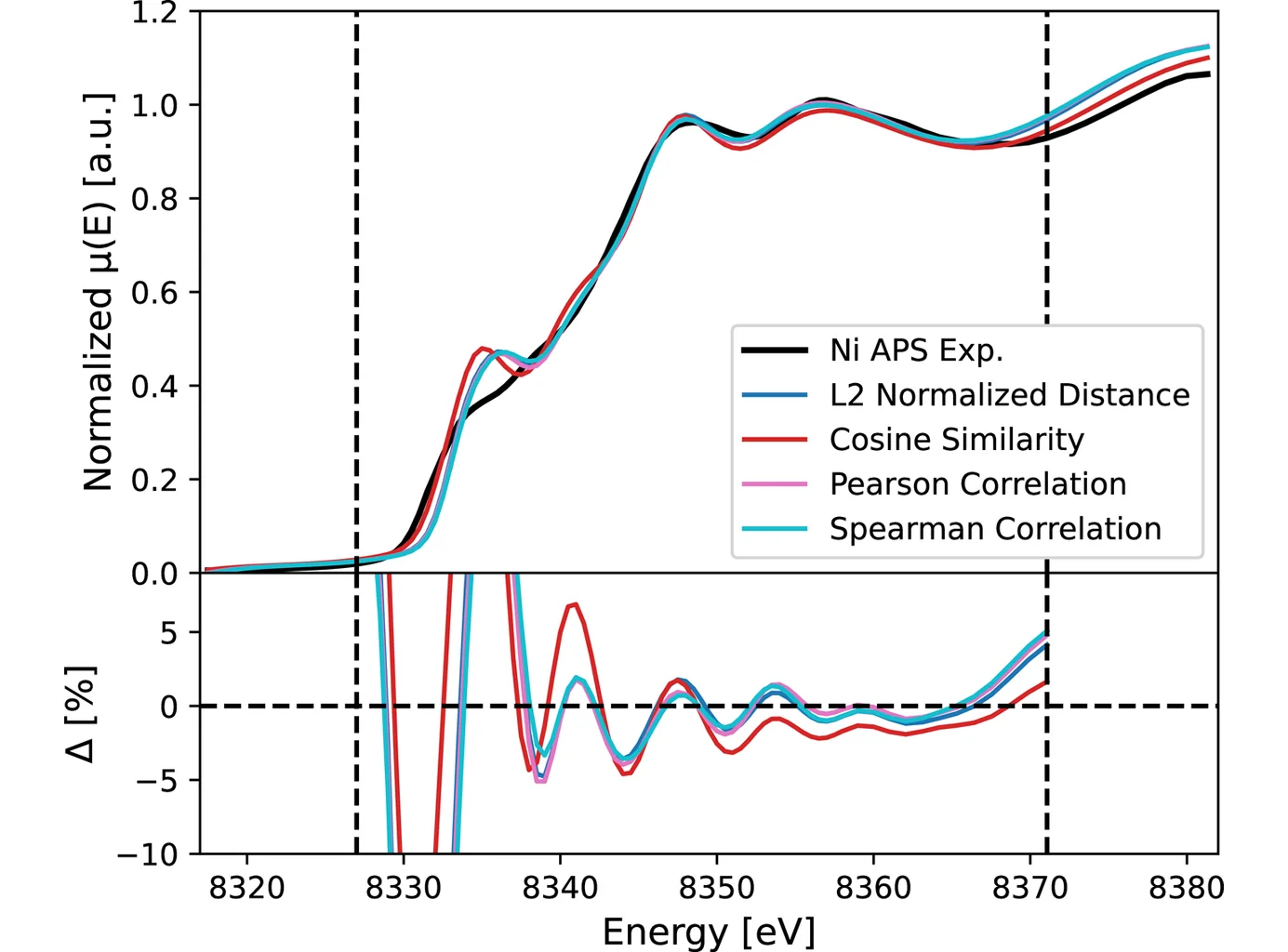
Results of approximating the experimental Ni APS spectrum using the *FDMNES* package. The upper section shows the edge-normalized experimental μ_E_ and simulated μ_T_ XANES spectra; the lower section shows the relative difference between the simulated spectrum and the experimental one, Δ = (μ_T_ − μ_E_)/μ_E_ × 100%. Vertical dashed lines denote the fitted energy range.

For every experimental spectrum, we performed an optimization procedure with both *FEFF* and *FDMNES* packages using objective functions based on different metrics: L2 normalized distance, cosine similarity, Pearson correlation coefficient, and Spearman rank correlation score. The cumulative results of optimized simulations for various metrics can be seen in Table 6 ▸ for the *FEFF* package and in Table 7 ▸ for the Ni APS sample simulated with *FDMNES*. To compare the metrics’ performance on a common basis, we provide the *R* factors for *FEFF* results in Table 8 ▸. The values were calculated between experimental spectra and theoretical spectra, integral-scaled to the experimental one. Fig. 2 ▸ shows the *FEFF* spectra optimized with different metrics against the experimental one, with the relative difference in the lower panel. The simulated spectra were energy-aligned and have their intensities integral-normalized. Due to limited computational resources, a similar comparison for the *FDMNES*-optimized simulation is shown in Fig. 3 ▸ for the Ni APS spectrum only.

As noted in Section 2.4, within the objective function, a secondary objective minimization procedure was applied to perform the energy alignment. In all cases, the main objective function was used as the minimization loss, except for Spearman rank correlation, which, due to its definition, is not sensitive to energy misalignment. In this case, we used L2 normalization distance as a secondary objective.

Despite the metrics chosen for this study being insensitive to the intensity of the spectral features, the objective functions were found to be non-convex, and the optimization results were not reproducible, even with secondary minimization based on the linear energy shift. A variation of the lattice constant was also tested to compensate for the energy scaling (*E*_scaling_) effect, but it did not result in any meaningful improvement. Therefore, we had to include *E*_scaling_ in addition to the linear energy correction. While such a correction is acknowledged as lacking physical grounds, it can be shown that it compensates for some of the systematic discrepancies of the model, helps to get the overall spectrum shape closer to the experimental and consistently reduces the residual, indicating it captures a systematic rather thana random discrepancy.

**Figure 4:**
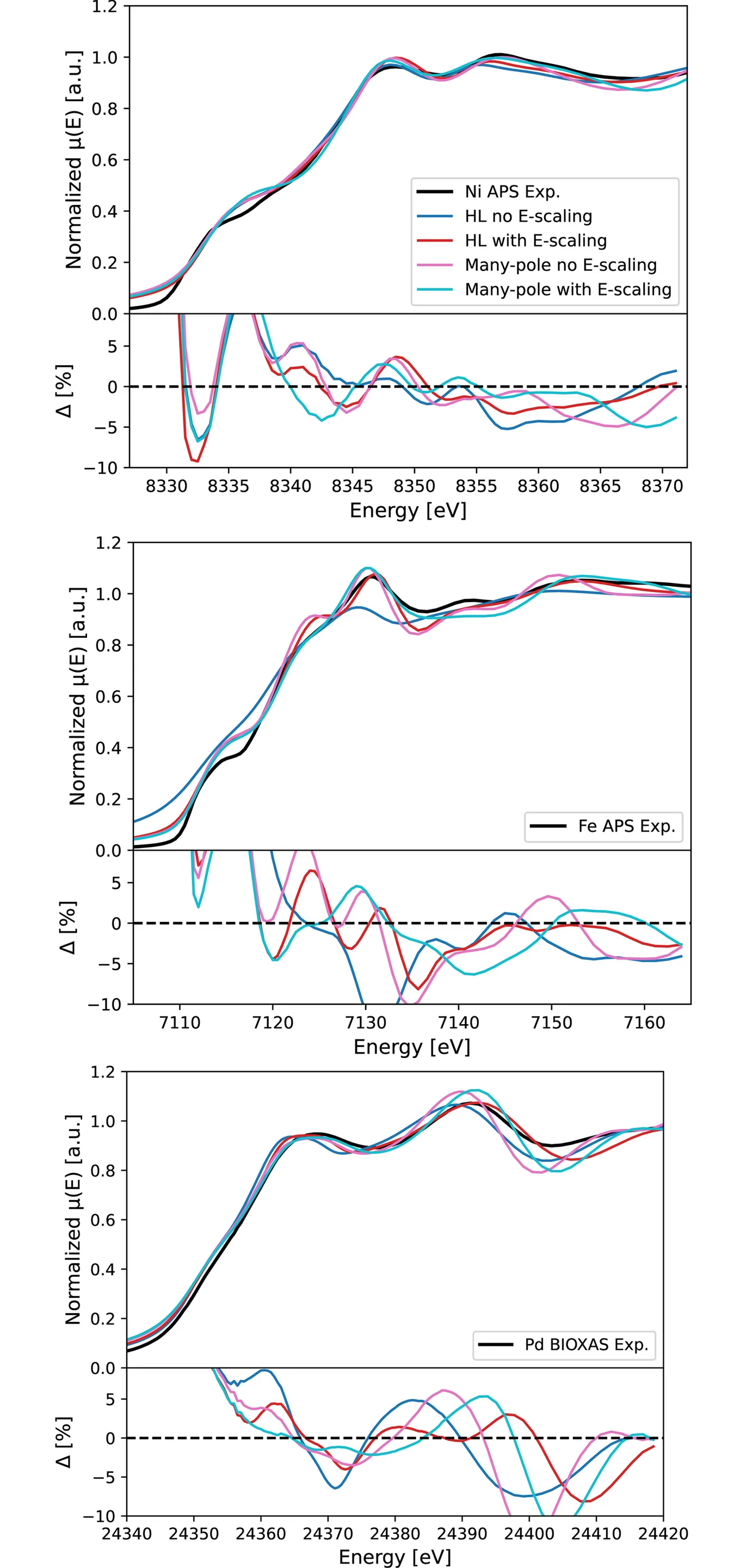
Effect of *E*_scaling_ and many-pole approximation on the *FEFF* simulation spectra with BO optimization using the L2 normalized distance objective function. The color legend in the top view applies to the two other views.

Table 9 ▸ shows L2 normalized distance and Spearman correlation metric values calculated for the simulated foil spectra optimized using baseline Hedin–Lundqvist (HL) and many-pole self-energy (MPSE) models with *E*_scaling_ enabled and disabled. The L2 metric is less uniform than the Spearman correlation because it is more weighted toward the edge-region intensity. The Spearman correlation shows that the spectrum shape modeling is improved when either *E*_scaling_ or a many-pole model is used. The plots in Fig. 4 ▸ also demonstrate this. The post-edge area has a smoother ratio of simulated spectrum to the experimental one if either *E*_scaling_ or the many-pole model is enabled, with the highest visual similarity achieved when both are enabled.

This observation lies in agreement with the *FEFF* documentation (Kas *et al.*, 2021 ▸), which recommends enabling the MPSE option for MPSE approximation ‘if peaks are slightly beyond the edge need to move to slightly higher energies and require more broadening’, which essentially happens when one applies the *E*_scaling_. This scaling thus compensates for the insufficient precision of the HL model, which can be improved with the many-pole approximation for two of the three considered foils (Ni, Pd). Numerically, it is reflected by the fact that switching from HL to the many-pole model changes the required *E*_scaling_ coefficient from 0.960 to 0.975 for Ni APS, from 0.930 to 0.914 for Fe APS and from 0.930 to 0.950 for Pd BIOXAS sample. The more feature-rich absorption edge potentially causes the issue with the iron spectrum.

**Figure 5:**
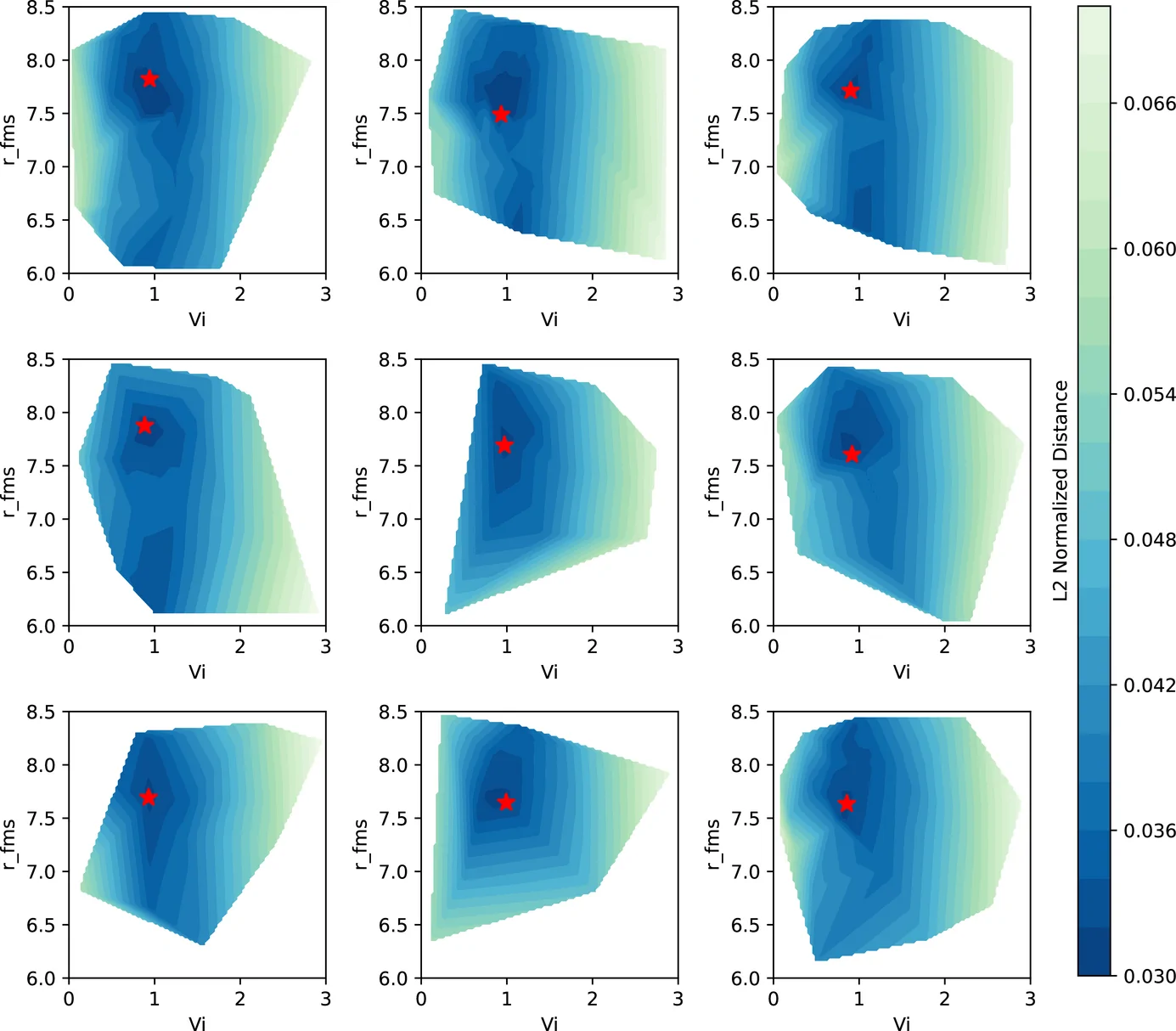
Objective function landscape plots calculated by the BO algorithm for the *FEFF* simulation parameters *V*_*i*0_ versus *R*_FMS_ for nine independent Ni APS sample fits with L2 normalized distance used as a metric. The stars denote the best parameter values found during the optimization.

To test the optimization convergence, we performed several runs with identical inputs but different initial random seeds. For example, fitting the Ni APS sample against L2 normalized distance metric gives good convergence of the resulting *R* factor *R*_*f*_ = 0.0304 ± 0.0004 with parameters *V*_*i*0_ = 0.90 ± 0.04 and *R*_FMS_ = 7.7 ± 0.1, and lower sensitivity for *R*_SCF_ = 6.7 ± 0.6. Fig. 5 ▸ provides a set of contour plots showing a similar loss function landscape for (*V*_*i*0_, *R*_FMS_) coordinates that were obtained in independent optimization runs.

**Figure 6:**
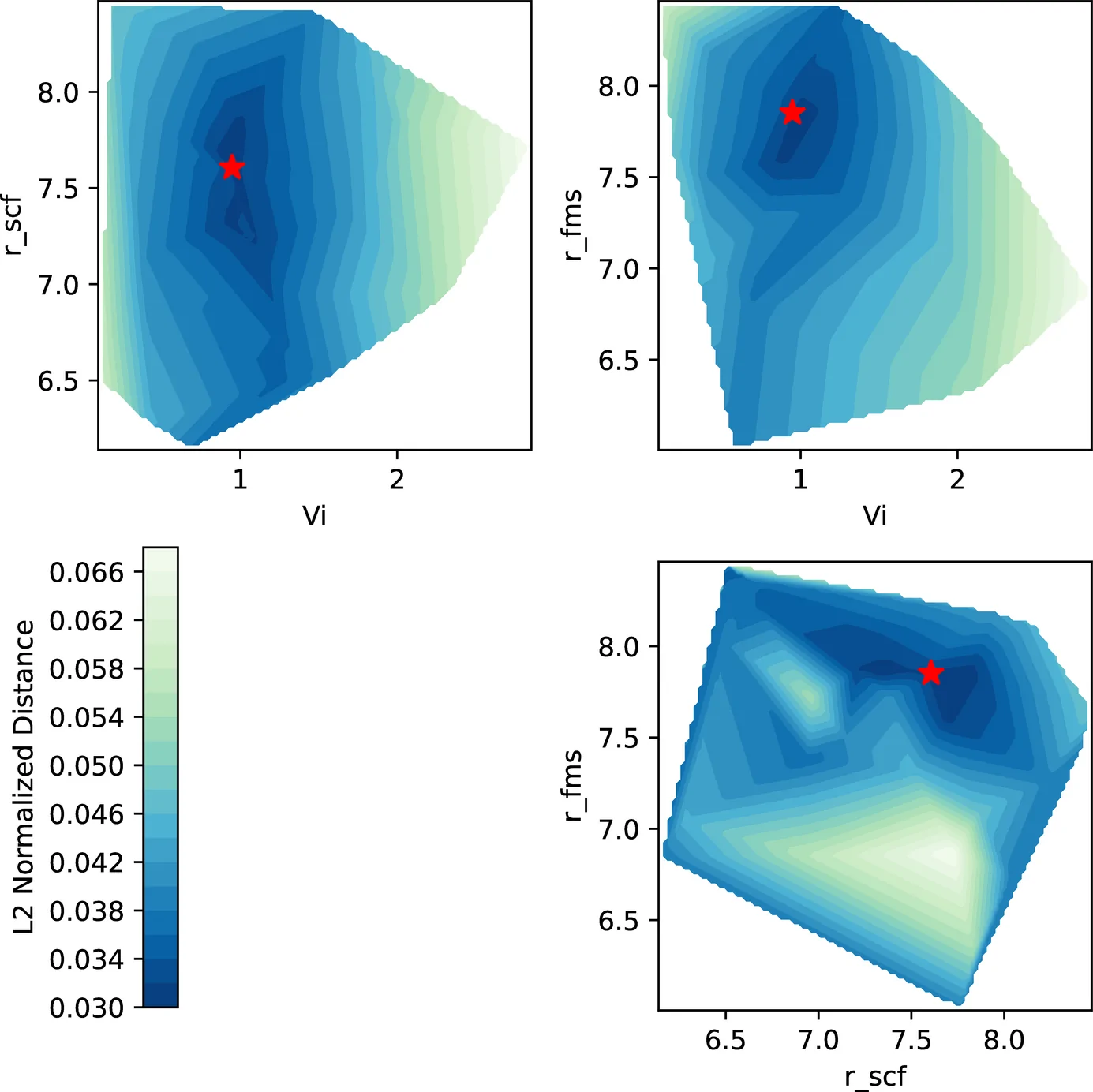
Objective function landscape plots for the BO algorithm for the Ni APS sample fitted with L2 normalized distance. The stars denote the best parameter values found during the optimization.

For this particular case of the Ni APS sample simulated with L2 normalized distance optimization, an example of contour plots for the complete set of parameters that were explored can be seen in Fig. 6 ▸. These figures show the 2D projection landscape of the objective function on the selected dimensions, with the *Z*-axis corresponding to the L2 normalized distance metric. From such a distribution, one can examine the convexity of the objective function relative to (*V*_*i*0_, *R*_SCF_) and (*V*_*i*0_, *R*_FMS_) parameter pairs. In the (*R*_FMS_, *R*_SCF_) projection, the objective function may have several local minima, which agrees with the observed low sensitivity to the *R*_SCF_ parameter (Fig. 8).

From the quantitative comparison of optimized results between *FEFF* and *FDMNES* for the Ni APS sample one can see that, for all metrics, *FEFF* provides better agreement than *FDMNES*. However, looking at the graphical spectra representation, one can see that the main contribution to the *FDMNES* discrepancy comes from an inaccurate description of resonant enhancement due to *p*–*d* states hybridization (mid-edge peak at 8335 keV), while the area after the white line shows better matching than *FEFF*.

**Figure 7:**
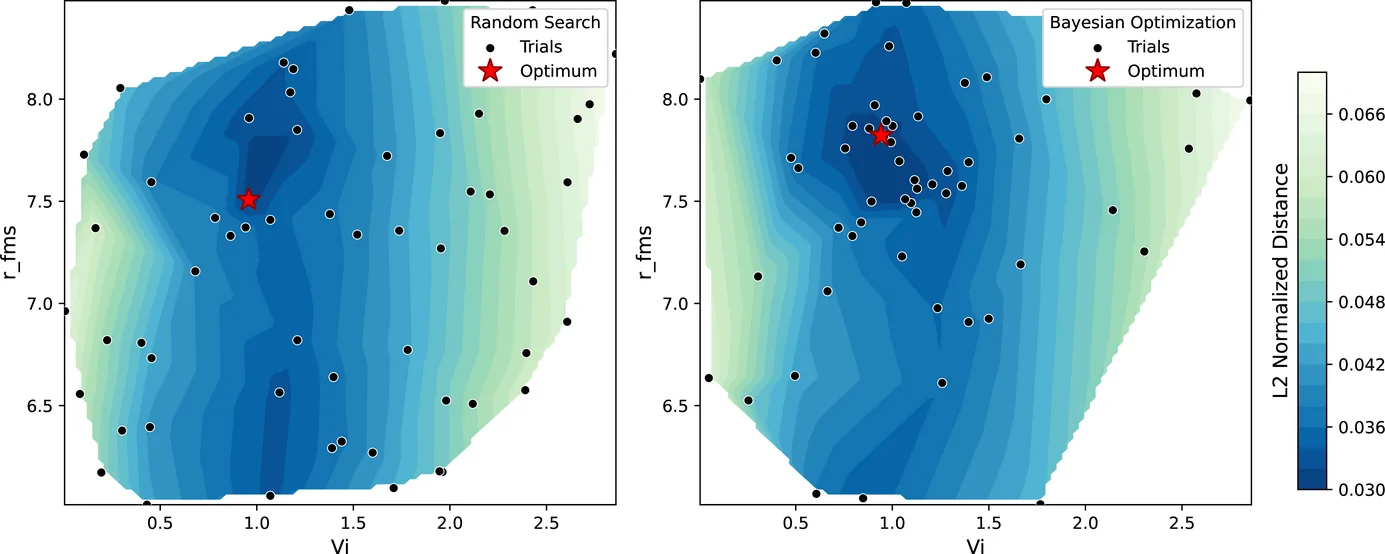
Comparison of random search (left) and BO (right) algorithm trials (black dots) while converging to the optimum (red star) plotted for Ni APS sample fitted with L2 normalized distance.

It is interesting to see how the BO algorithm performs in comparison with the random search method. In this test, we compare the average number of trials that both algorithms require to converge to the same optimum, given the same non-improvement tolerance of ten runs and the same parameter space. This quantity is calculated for BO repeated runs directly: the 〈L2〉 = 0.0310 ± 0.0004 minimum value of the L2 objective function is reached on average in 17 runs for the *FEFF* code when simulating the Ni APS sample. For the random sampling, we deduce this quantity from computing the probability *p* of reaching the 95% confidence interval of the same optimum. Over the series of 1000 simulations with randomized parameter values within the parameter set, 25 outcomes have reached this limit, which gives *p* = 0.025. According to the Bernoulli distribution, the expected number of trials before success is 1/*p* = 40. Adding the non-improvement tolerance of ten trials, we obtain that in this particular example the BO reaches the optimum on average about three times faster than the random sampling. Fig. 7 ▸ demonstrates how BO is more efficient in converging to the optimum value relative to the random search algorithm.

## 4. Discussion

**Figure 8:**
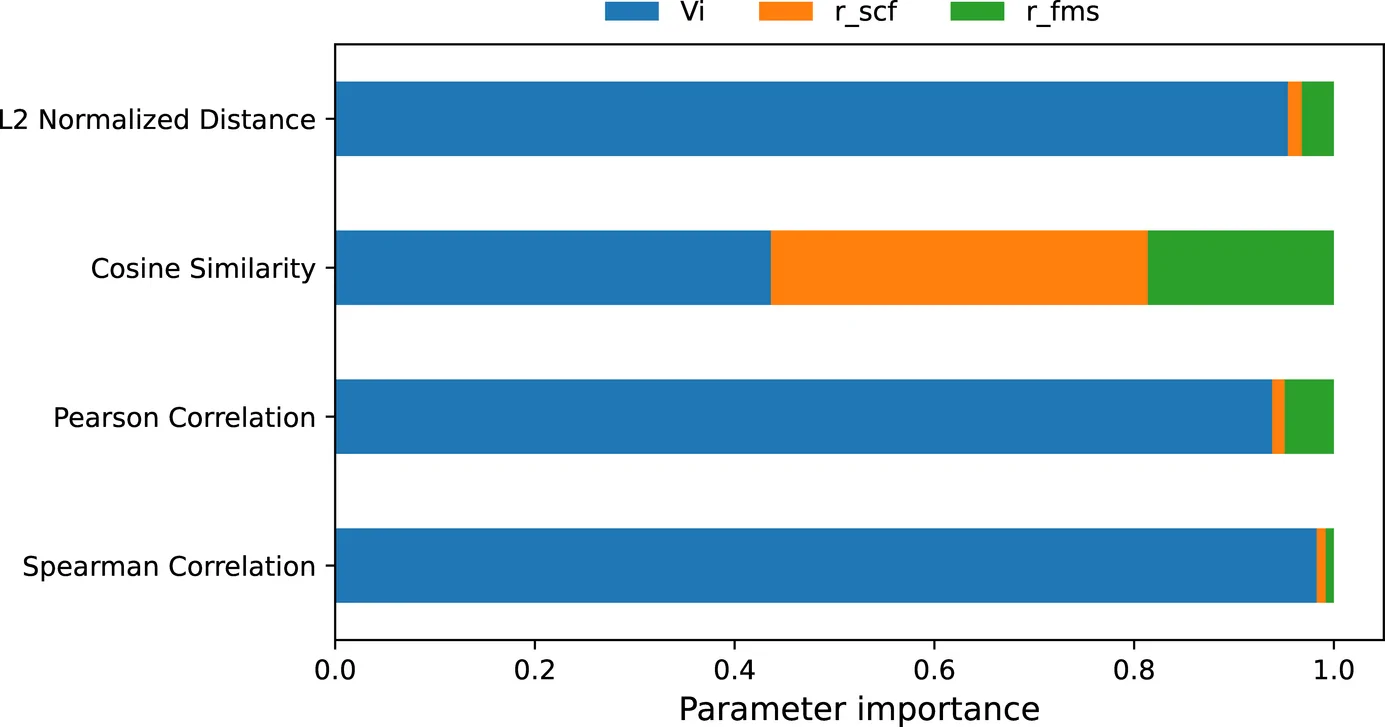
Optimization parameter sensitivity (importance) calculated for Ni APS sample and different metrics.

The sensitivity of optimization to the simulation parameters can be characterized by the so-called parameter importance. In the case of the BO algorithm implemented in the Optuna library, they are defined by how much each parameter contributed to the variation in the objective value after the optimization procedure (Hutter *et al.*, 2014 ▸). Fig. 8 ▸ shows the parameter importance values calculated for the Ni APS sample when using different objective functions. We see that the sensitivity to the *V*_*i*0_ parameter prevails in the fitting procedure for most of the objective functions, but particularly for cosine similarity *R*_SCF_ and *R*_FMS_ also become significant.

**Figure 9:**
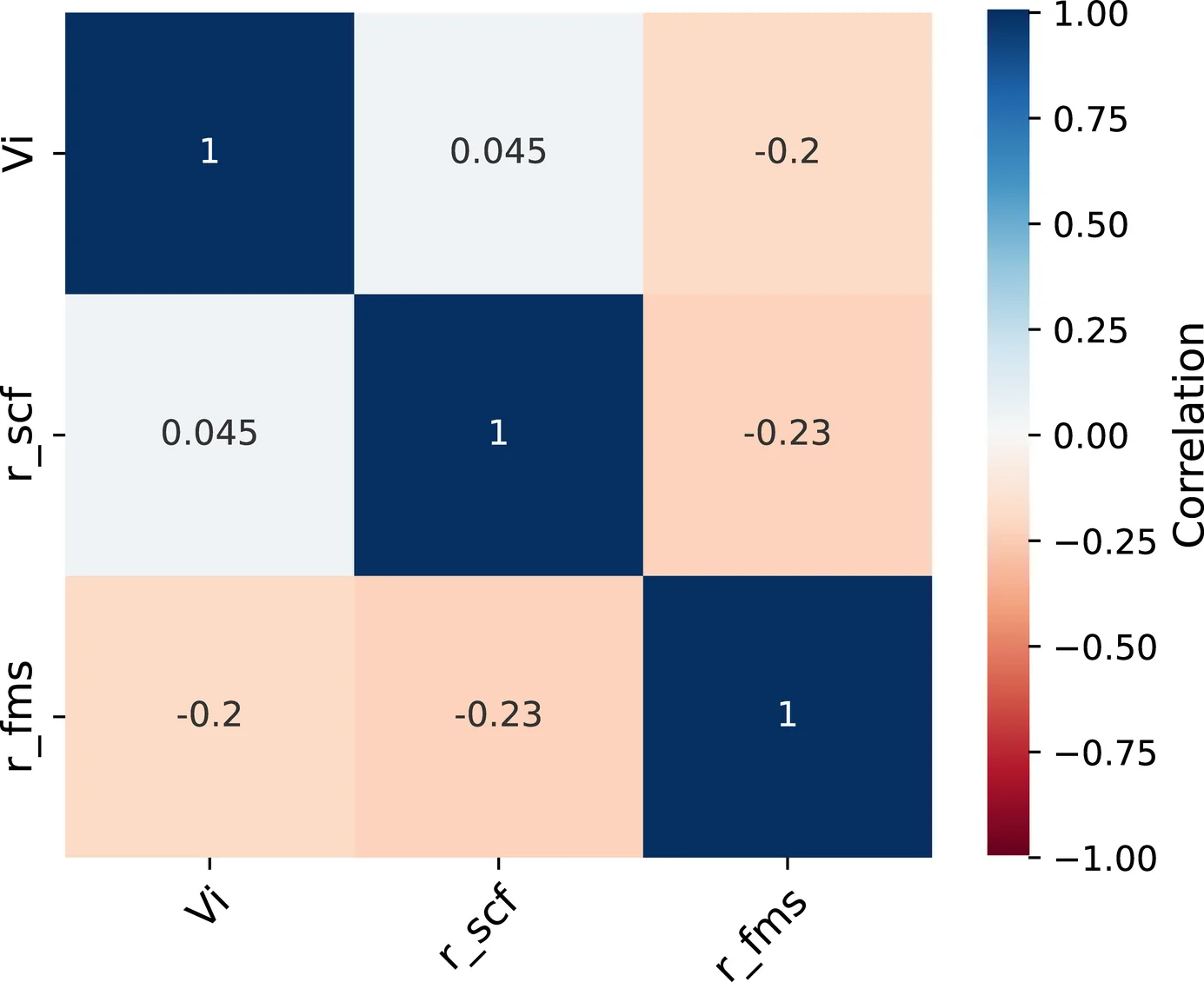
Correlations between optimization parameters for the Ni APS sample fitted with the L2 metric.

It is important to note that these quantities do not necessarily reflect the parameter relevance, and one should not rely on their values to decide whether to include or discard the parameters from the optimization procedure. The objective function landscape serves better for this purpose. We also find it valuable to check the parameter correlations because they have a direct impact on the reproducibility of the results. Fig. 9 ▸ shows the heatmap of correlation coefficients for the Ni APS sample fitted with *FEFF* simulation using the L2 metric. One can see that the correlation values between *V*_*i*0_, *R*_SCF_ and *R*_FMS_ parameters are below ∼0.2, which can be considered as negligible.

As expected the BO algorithm, when applied for fitting of experimental XANES spectra, is more resource-effective as compared with explicit grid-search or random search approaches. Both *FEFF* and *FDMNES* calculations can be time-consuming, and reducing the number of iterations required for the optimization procedure is always beneficial. The definition of agreement to the experimental spectra is, however, ambiguous, and the final result is heavily dependent on the chosen metric. The commonly used *R* factor and L2 normalized distance can give a closer quantitative match, but the correlation-based metrics seem to provide better shape-wise agreement, which complies with the fingerprinting logic. One should also note that the spectrum similarity objective function is non-convex by default, and one may need to apply energy transformation for the simulated spectra of some materials. The linear energy transformation applied in this work helps to achieve robustness of the optimization while preserving the spectral features.

Note that there are alternative methods to improve the theoretical description of experimental spectra: apart from full-scale DFT calculations, one can improve *FEFF* results by enabling the MPSE model (Kas *et al.*, 2007 ▸) and provide an accurate calculation of the corresponding loss function. While deepening the domain knowledge is always beneficial, many aspects of theory may still be hidden from ordinary users as they will treat the simulation frameworks as a black box. The BO method can thus be used to partially compensate for such shortcomings. In addition, the XANES BO may be efficiently applied to the structural and/or chemical composition analysis, and, presumably, the structural disorder analysis (Li *et al.*, 2021 ▸).

Unlike other applications of Bayesian approaches to XAS analysis, which primarily help to decompose and interpret the spectra, our approach is targeted at obtaining the best possible results when using *abinitio* calculations that are based on prior knowledge of the material structure.

While the current state of the XANES simulation brings it extremely close to the experiment, its application to numerical analysis is still limited. The *abinitio* codes are mostly used for qualitative assessment of trends and tendencies that can be observed due to structural or chemical changes. Having a method for more precise configuration of *FEFF* or *FDMNES* should make numerical analysis more feasible by allowing a direct matching between data and simulation.

## 5. Conclusion

Using the BO algorithm based on L2 normalized distance, cosine similarity, Pearson correlation coefficient, Spearman rank correlation score objective functions we performed optimization of input parameters for theoretical XANES calculations by *FEFF* and *FDMNES* in order to achieve the best match between calculated and experimental reference spectra. The imaginary optical potential correction and full multiple scattering radius parameters were found to have the strongest influence on the spectra, with the most optimal values being different for every material. For example, for Ni *K*-edge transmission spectrum the optimal *FEFF* parameters are *V*_*i*0_ = 0.95, *R*_FMS_ = 7.9 and *R*_SCF_ = 7.6 when using L2 normalized distance and *V*_*i*0_ = 1.5, *R*_FMS_ = 6 and *R*_SCF_ = 7.5 when using Spearman correlation. This is another valuable finding: different objective functions give different optima. One may choose the correlation-based metrics if they want more uniform shape agreement (see, for example, the relative difference plots in Fig. 2 ▸). We observe that applying the BO procedure gains a significant time reduction relative to the random search optimization. The resulting optimal parameter sets are intended to be used for modeling spectra of transition metal species often predicted theoretically (such as bi-, tri-metallic compounds, high entropy alloys, *etc*.), but which are difficult to obtain experimentally as reliable, well defined reference samples.

Given the promising results of tuning the XANES simulations using the BO algorithm, we can further extend its application to actual catalysis phenomena and energy storage research. This includes local atomic structure, surface versus bulk contributions and structural heterogeneity and disorder under realistic operation conditions. The most interesting application of the present work would be tests of the quantitative interpretation of XANES spectra—a field where the simulation-based methods are currently limited in terms of precision.

## Figures and Tables

**Figure 10:**
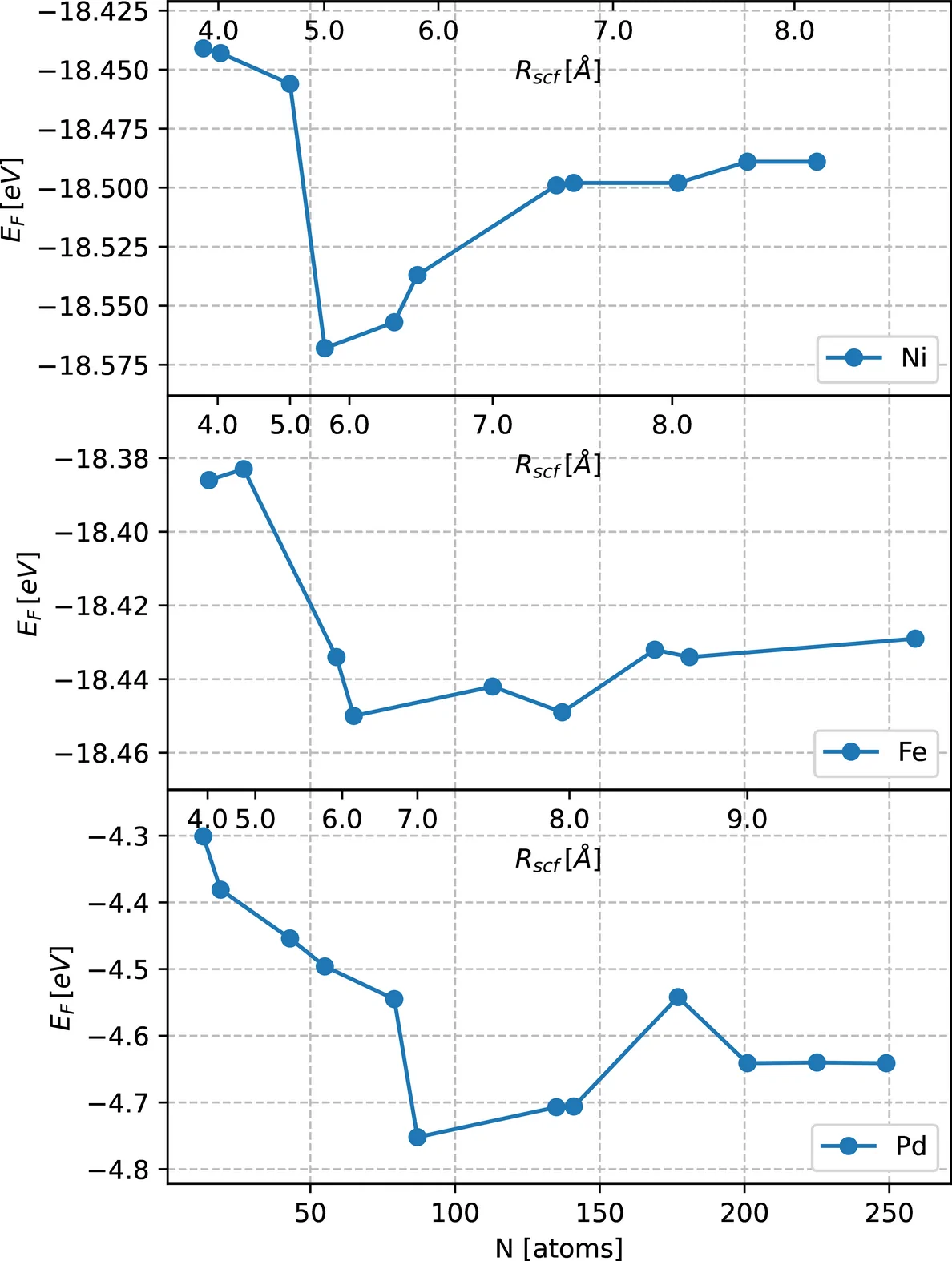
SCF radius convergence test with *FEFF* calculations for Ni, Fe and Pd foils. The convergence values start for clusters around above 100 atoms.

**Table 1:** Selected *FEFF* (v10.0.0) configuration parameters relevant to simulations of XANES and EXAFS spectra of metallic foils *d*_NN_ corresponds to the distance to the nearest neighboring atom.

Card	Parameter	Role	Region	Type	Units	Default	Range
S02	s02	Amplitude reduction factor	EXAFS	Float	–	1.0	0 to ∼1
FMS	rfms	Full multiple scattering radius	XANES	Float	Å	–	≥ 0
SCF	rfms1	Self-consistency cluster radius	XANES	Float,	Å	–	≥ 0
EXCHANGE	ixc	Fine structure model	XANES/EXAFS	Boolean	–	0	0 to 5
EXCHANGE	vr0	Fermi level correction	XANES/EXAFS	Float	eV	0	−∞ to ∞
EXCHANGE	vi0	Optical potential correction	XANES/EXAFS	Float	eV	0	≥ 0
RPATH	rpath	Maximum effective path length	EXAFS	Float	Å	2.2 × *d*_NN_	> 0

**Table 2:** Selected *FDMNES*/*FDMX* (v2025_07_11) parameters relevant to simulations of XANES and EXAFS spectra of metallic foils

Keyword	Role	Region	Type	Units	Default	Range
Radius	Cluster size	XANES/EXAFS	Float	Å	–	> 0
R_self	SCF cluster radius	XANES/EXAFS	Float	Å	min(Radius, 3.5)	> 0
Eimag	Energy imaginary part	XANES	Float	eV	0.1	> 0
E_cut	Suppress transitions below it	EXAFS	Float	eV	–	−∞ to ∞

**Table 3:** Samples of monometallic transmission XAS spectra measurements used in the analysis

Sample	Facility	Beamline	Database	Sample id	Reference
Ni APS	APS	13-ID-C,D	XASLIB	Ni_metal_rt_01	Newville (2001 ▸)
Ni P65	PETRA III	P65	RefXAS	00bc0e2b-0fd7-4eac-bd86-5c642b11082f	Vajglová *et al.* (2023 ▸)
Fe APS	APS	13-BM-D	XASLIB	Fe_metal_rt_01	Newville (2002 ▸)
Fe P65	PETRA III	P65	RefXAS	8ee33eb4-9cf4-4550-a0b8-1eba1e0fdd09	Vajglová *et al.* (2023 ▸)
Pd BIOXAS	CLS	BIOXAS-S	CLS XASDB	kle4ny	Spasyuk *et al.* (2025 ▸)
Pd P65	PETRA III	P65	RefXAS	e711cd2b-051d-4d43-a579-885d2562c4e9	Selinsek *et al.* (2018 ▸)

**Table 4:** Edge-step normalization parameters that were used for experimental spectra preprocessing

Element	Pre-edge range	Post-edge range	Post-edge polynomial degree
Ni	(−150, −40)	(290, 700)	2
Fe	(−150, −40)	(200, 700)	2
Pd	(−170, −90)	(250, 600)	2

**Table 5:** Simulation parameter limits for BO

Package	Parameter	Notation	Min	Max
*FEFF*	vi0	*V*_*i*0_	0	3
	rfms	*R*_FMS_	6	8.5
	rfms1	*R*_SCF_	6	8.5
*FDMNES*	Radius	*R*	6	8.5
	R_self	*R*_self_	6	7.5
	Delta_E_conv	Δ*E*_conv_	−3	3
	Eimag	*E*_imag_	0	3

**Table 6:** *FEFF* parameters tuned to simulate given sample using different objective functions The ‘Value’ column stands for the corresponding metrics.

Objective	Sample	Value	*V*_*i*0_	*R*_SCF_	*R*_FMS_
L2 normalized distance	Ni APS	0.031	0.95	7.6	7.9
	Ni P65	0.029	0.9	6.6	7.7
	Fe APS	0.048	0.88	8.1	8
	Fe P65	0.031	0.68	7.4	6
	Pd BIOXAS	0.035	0.0025	7.7	6.9
	Pd P65	0.033	0.014	6.8	6.9
Cosine similarity	Ni APS	0.00025	0.85	7.5	7.7
	Ni P65	0.0002	0.97	6.5	7.8
	Fe APS	0.00056	0.83	8.2	7.6
	Fe P65	0.00022	0.58	6.4	6.1
	Pd BIOXAS	0.00029	0.058	7.2	7.2
	Pd P65	0.00031	0.0034	8	7.9
Pearson correlation	Ni APS	0.00068	1.4	7.8	6.1
	Ni P65	0.00072	1.4	6.7	8
	Fe APS	0.00064	1.7	7.7	7.2
	Fe P65	0.00074	1.8	7.4	7.4
	Pd BIOXAS	0.0011	1	7.4	8.5
	Pd P65	0.0018	0.42	7.5	6.9
Spearman correlation	Ni APS	0.0019	1.5	7.5	6
	Ni P65	0.0025	1.5	6.8	6
	Fe APS	0.002	1.5	7.8	6.4
	Fe P65	0.003	1.4	6.9	6.5
	Pd BIOXAS	0.023	0.48	7.2	6.9
	Pd P65	0.029	0.025	7.2	8.4

**Table 7:** *FDMNES* parameters tuned to simulate Ni APS sample using different objective functions ‘Value’ column stands for the corresponding metrics.

Objective	Sample	Value	*R*	*R*_self_	Δ*E*_conv_	*E*_imag_
L2 normalized distance	Ni APS	0.042	7.6	6.9	1.8	0.032
Cosine similarity	Ni APS	0.00048	7	6	−0.64	0.099
Pearson correlation	Ni APS	0.0023	7.8	7.5	1.2	0.068
Spearman correlation	Ni APS	0.0071	7.7	6.4	−2.1	0.21

**Table 8:** *R* factors calculated for *FEFF* spectra optimized to different samples using selected metrics

Objective	Ni APS	Ni P65	Fe APS	Fe P65	Pd BIOXAS	Pd P65
L2 normalized distance	0.031	0.027	0.045	0.028	0.032	0.044
Cosine similarity	0.031	0.027	0.045	0.026	0.031	0.046
Pearson correlation	0.036	0.033	0.059	0.045	0.044	0.048
Spearman correlation	0.037	0.033	0.063	0.046	0.035	0.043

**Table 9:** L2 normalized distance and Spearman correlation coefficient metrics calculated post-hoc for *FEFF* spectra across four simulation configurations (*E*_scaling_  ×  self-energy approximation) optimized with L2 objective HL is Hedin–Lundqvist.

		HL no *E*_scaling_	HL with *E*_scaling_	Many-pole no *E*_scaling_	Many-pole with *E*_scaling_
Ni APS	L2	0.0379	0.0322	0.0391	0.0361
	Spearman	0.0156	0.0101	0.0059	0.0034
	*E*-scale	–	0.9604	–	0.9747
Fe APS	L2	0.1003	0.0477	0.0574	0.0417
	Spearman	0.0271	0.0034	0.0106	0.0050
	*E*-scale	–	0.9304	–	0.9142
Pd BIOXAS	L2	0.0489	0.0352	0.0522	0.0446
	Spearman	0.0429	0.0223	0.0192	0.0078
	*E*-scale	–	0.9304	–	0.9496

## Data Availability

The data and the computer programs supporting the reported results can be shared upon a reasonable request.

## Appendix A Similarity metrics for spectrum comparison

To quantify the agreement between experimental and theoretical spectra in the procedure of optimizing theoretical parameters in *FEFF* and *FDMNES* packages, we employed several metrics which capture various aspects of spectral similarity. Let the intensities of the experimental (exp) and theoretical (th) spectra, evaluated on the same energy grid of length *N*, be denoted as *I*_exp_ = (*I*_exp, 1_, …, *I*_exp, *N*_) and *I*_th_ = (*I*_th, 1_, …, *I*_th, *N*_).

### A1. L2 normalized distance

The L2 normalized distance quantifies the shape dissimilarity between two spectra after each is vector-normalized to unit Euclidean norm. It is defined as

This metric is sensitive to differences in spectral shape, while being invariant to absolute scaling.

### A2. *R* factor

The *R* factor is commonly used in EXAFS analysis and measures the relative deviation between the spectra,

It captures the pointwise agreement in intensities, vector-normalized to the total signal of the experimental spectrum.

### A3. Cosine similarity

Cosine similarity measures the cosine of the angle between two vectors in high-dimensional space,

It is a shape-based metric, insensitive to offset and absolute scaling.

### A4. Pearson correlation coefficient

Pearson correlation coefficient quantifies the linear correlation between two spectra,

where  and  denote the mean intensity of each spectrum. This metric reflects how linearly the intensities vary with each other, overall shape similarity and amplitude accuracy.

### A5. Spearman rank correlation

Spearman rank correlation coefficient assesses the degree to which the relationship between spectra is monotonic. It is computed as

where *d*_*i*_ = rank(*I*_exp, *i*_) − rank(*I*_th, *i*_) is the difference between the ranks of the intensities. This metric is robust to nonlinear intensity transformations and emphasizes the ordinal agreement of spectral features. It is thus more focused on the order in which the spectrum features appear along the energy axis (*e.g.* peak positions, pre- and post-edge).

## Appendix B SCF convergence

In order to validate the SCF convergence in XAS simulation, the manuals recommend observing the convergence of the Fermi level energy. The general recommendation (Moreno *et al.*, 2007 ▸) is to observe the stability between iterations within one run and to pick *R*_SCF_ values from the plateau region. Fig. 10 ▸ shows an example of the SCF convergence test for Ni, Fe and Pd monometallic crystal simulations studied in this work. It is visible that, for Ni and Fe, the approximate stability is reached for *R*_SCF_ > 6.5, corresponding to cluster size >100 atoms. For Pd, the limits are even higher, and start from *R*_SCF_ > 9 (150 atoms), which makes iterative calculations very expensive.
